# Characterizing imaging radiation risk in a population of 8918 patients with recurrent imaging for a better effective dose

**DOI:** 10.1038/s41598-024-56516-1

**Published:** 2024-03-14

**Authors:** Francesco Ria, Madan M. Rehani, Ehsan Samei

**Affiliations:** 1 Center for Virtual Imaging Trials, Carl E. Ravin Advanced Imaging Labs, Clinical Imaging Physics Group, Departments of Radiology, Duke University Health System, 2424 Erwin Road, Suite 302, Durham, NC 27710 USA; 2 Radiology Department, Massachusetts General Hospital, 175 Cambridge Str. Suite 0244, Boston, USA; 3 Center for Virtual Imaging Trials, Carl E. Ravin Advanced Imaging Labs, Clinical Imaging Physics Group, Medical Physics Graduate Program, Departments of Radiology, Physics, Biomedical Engineering, and Electrical and Computer Engineering, Duke University, 2424 Erwin Road, Suite 302, Durham, NC 27710 USA

**Keywords:** Computed tomography, Risk factors

## Abstract

An updated extension of effective dose was recently introduced, namely relative effective dose (${E}_{r}$), incorporating age and sex factors. In this study we extended ${E}_{r}$ application to a population of about 9000 patients who underwent multiple CT imaging exams, and we compared it with other commonly used radiation protection metrics in terms of their correlation with radiation risk. Using Monte Carlo methods, ${E}_{r}$, dose-length-product based effective dose (${E}_{DLP}$), organ-dose based effective dose (${E}_{OD}$), and organ-dose based risk index (${\text{RI}}$) were calculated for each patient. Each metric’s dependency to ${\text{RI}}$ was assessed in terms of its sensitivity and specificity. ${E}_{r}$ showed the best sensitivity, specificity, and agreement with ${\text{RI}}$ (R^2^ = 0.97); while ${E}_{DLP}$ yielded the lowest specificity and, along with ${E}_{OD}$, the lowest sensitivity. Compared to other metrics, ${E}_{r}$ provided a closer representation of patient and group risk also incorporating age and sex factors within the established framework of effective dose.

## Introduction

One of the leading priorities in radiological care is effectual use of technology to mitigate and minimize radiation risk. Traditionally, radiation risk in radiological exams has been assessed in terms of device output quantities (tube current, CTDI_vol_, KAP, etc.)^1,2^. Such quantities, however, represent only the radiation *input* to the patient and cannot be directly related to patient risk. In particular, moving from device radiation outputs to dose and risk requires the inclusion of patient specific information such as patient body habitus^3,4^. Moreover, as radiation detriment is strongly related to patient age and sex, such attributes should be included in any accurate risk estimation^5^. Current metrics of radiological detriments have remained patient-generic, even though there is a great desire and relevance to make such assessments patient-specific.

To address this need, the International Commission of Radiological Protection (ICRP) developed effective dose ($E$), initially defined for occupational exposures based on an idealized human model and extended to medical exposures in defined conditions^6,7^. $E$ is ideally calculated through estimation of organ doses, which tends to be complex. However, approximation methods have been developed to facilitate the calculation of $E$. In particular, in Computed Tomography, $E$ can be estimated by applying anatomic region conversion factors to the dose length product (DLP) producing the so-called DLP-based effective dose (${E}_{DLP}$). It has been shown that ${E}_{DLP}$ can approximate the organ dose-based effective dose (${E}_{OD}$)^2^. But this approximation does not hold when the commonly-used tube current modulation (TCM) is in effect^8^. Further, neither current ${E}_{DLP}$ nor ${E}_{OD}$ account for the patient sex and age.

A potential solution to this challenge is proposed in the recent ICRP publication 147: “Organ and tissue absorbed doses are now calculated using male and female phantoms of the human body for children of various ages as well as for adults. A consistent approach would be to calculate the corresponding detriment and relative detriment values as well, and calculate effective dose coefficients using these values. Averaging for all workers and all members of the public could then be done as a final stage, or dose criteria could be set with reference to the range of effective dose coefficients and detriment values presented. This approach would not affect the practical application of the system of protection in general terms, but would facilitate consideration of appropriate protection for population subgroups”^9^. As an implementation of this solution, a recent study proposed relative effective dose (${E}_{r}$) that incorporates age- and sex-factors^10^. However, the method was tested only in a small patient dataset.

The purpose of this study was to assess the application of ${E}_{r}$, as well as ${E}_{OD}$ and ${E}_{DLP}$, to a cohort of patients who underwent recurrent imaging associated with cumulative ${E}_{OD}$ over 100 mSv and to compare it with the closest surrogate of patient specific radiation risk, risk index^5^.

## Material and methods

This study was performed in compliance with the Health Insurance Portability and Accountability Act (HIPAA), it was approved by the Institutional Review Board (IRB) at Massachusetts General Hospital, and informed consent waiver was obtained. The work involved retrospective analysis radiation dose records that patients received without any bearing on patient's diagnosis or treatment and there were no experiments on humans and/or the use of human tissue samples. The study included 8918 patients (4311 female; 4607 male; median age: 71.2 year old; min age: 21.6 year old; max age: 99.98 year old) who underwent multiple CT imaging exams over 5 years at a major tertiary care hospital in USA between 2013 and 2017 (Table 1). Patient’s age and sex distributions reflected the considered clinical scenario.

**Table 1:** Age and sex distribution for the patients involved in the study.

Age (years)	Female	Male	Total
20–29	21	30	51 (0.6%)
30–39	129	152	281 (3.2%)
40–49	261	211	472 (5.3%)
50–59	538	584	1122 (12.6%)
60–69	1104	1106	2210 (24.8%)
70–79	1279	1391	2670 (29.9%)
80–89	757	930	1687 (18.9%)
90 >	222	203	425 (4.8%)
Total	4311 (48.3%)	4607 (51.7%)	8918

A dose monitoring system (Radimetrics, Bayer HealthCare, Leverkusen, Germany) provided patient information, age, sex, and scanner radiation output in terms of DLP^11^. The system also calculated with Monte Carlo methods organ doses (${OD}_{T}$) for 25 organs ($T$). Several metrics were calculated to perform the analysis (Table 2). The DLP-based effective dose (${E}_{DLP}$) was calculated multiplying the DLP by the anatomical region conversion factors for adult patients reported in Table A.2 of the ICRP publication 102^1^. Moreover, following the ICRP publication 103 tissue weighting factors, ${E}_{OD}$ was calculated per each patient^6^. The radiation risk index ($RI$) was estimated by applying the sex- and age- specific lifetime attributable risk of cancer incidence in the US population for the tissue $T$ (${r}_{T}$) reported in BEIR VII to the patient-specific organ doses ($OD_{T}$): $RI = \mathop \sum \limits_{T} r_{T} OD_{T}$^5,12,13^. Lastly, the updated relative effective dose ${(E}_{r})$ was calculated adjusting ${E}_{OD}$ for an age and sex patient-specific factor $f=\frac{RI}{{RI}_{rp}}$, where ${RI}_{rp}$ is the risk index calculated for a 35-year-old reference patient considering sex-averaged ${r}_{T}$ coefficients: $E_{r} = f \times E_{OD}$^10^. We tested the null hypotheses of no relationship between ${E}_{DLP}$, ${E}_{OD}$, and ${E}_{r}$, and $RI$, each. For each metric, mean, median, range, and standard deviation were further calculated.

**Table 2:** List of the risk metrics.

Metric	Name	Description	Unit
${E}_{DLP}$	DLP-based effective dose	Effective dose to a reference phantom based on CT radiation output and pre-calculated coefficients^1^	mSv
${E}_{OD}$	Organ dose-based effective dose	Effective dose based on patient organ doses for a specific imaging condition^6^	mSv
$RI$	Risk index	Risk index based on patient organ doses for a specific imaging condition^13^	Number of cancers per 100 patients
${RI}_{rp}$	Risk index for a reference patient	Risk index for 35 year old patient undergoing the same exam^13^	Number of cancers per 100 patients
$f$	f-factor	Risk index to relative effective dose conversion factor: $f=\frac{RI}{{RI}_{rp}}$	Dimensionless
${E}_{r}$	Relative effective dose	Effective dose based on patient organ doses for a specific imaging condition and adjusted for a factor that incorporates age- and sex- specific risk^10^	mSv’

Because risk index is considered the closest metric to patient risk, a linear regression was applied to assess each metric’s dependency to $RI$ assuming null intercept. The relative sensitivity of ${E}_{DLP}$, ${E}_{OD}$, and ${E}_{r}$ to the estimated risk was calculated in terms of a Risk Sensitivity Index (RSI) computed as a normalized fit slope by the ratio of the mean value of each metric and that of $RI$. Metrics with RSI values closer to unity better characterize the patient radiation dose. Lastly, to assess how well a metric can represent specific differences in radiation risks, the specificity, a Risk Differentiability Index (RDI) was calculated as the root mean square error (RMSE) of each fit divided by the fit slope. Metrics with RDI values closer to zero offer better differentiability. All data generated and analyzed in the study are available from the corresponding author on reasonable request.

## Results

**Figure 1:**
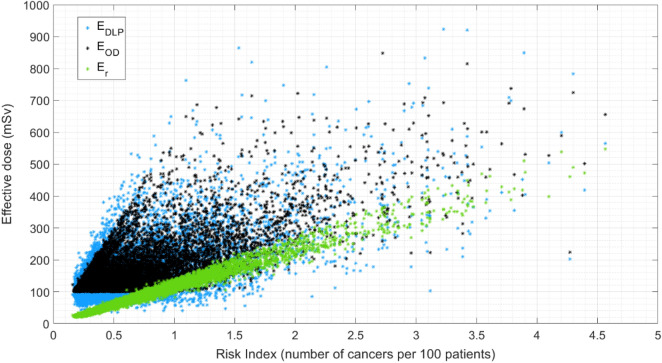
Distributions of E_DLP_, E_OD_, and E_r_ with respect of RI.

The significance test returned *p* values smaller than 0.01, rejecting the null hypotheses, implying that there is a significant relationship between ${E}_{DLP}$, ${E}_{OD}$, and ${E}_{r}$, and $RI$. Table 3 reports mean, median, range, and standard deviation for each metric, and the related distributions are plotted in Fig. 1. Consistently with patient cohort inclusion criteria, ${E}_{OD}$ distribution did not show data points below 100 mSv. However, the calculation of ${E}_{DLP}$ and ${E}_{r}$ for the same patients returned values lower than 100 mSv.

**Table 3:** Mean, median, range, and standard deviation for all metrics included in the study.

Metric	Mean	Median	Min–Max	Standard deviation
E_DLP_	166.1	140.7	34.8–923.1	91.8
E_OD_	176.4	146.9	100.0–848.0	86.8
RI	0.7	0.6	0.2–4.6	0.5
RI_rp_	1.5	1.3	0.6–7.9	0.7
f	0.5	0.4	0.2–2.1	0.2
E_r_	83.3	66.6	19.2–547.6	60.8

Units are described in Table 2.

**Figure 2:**
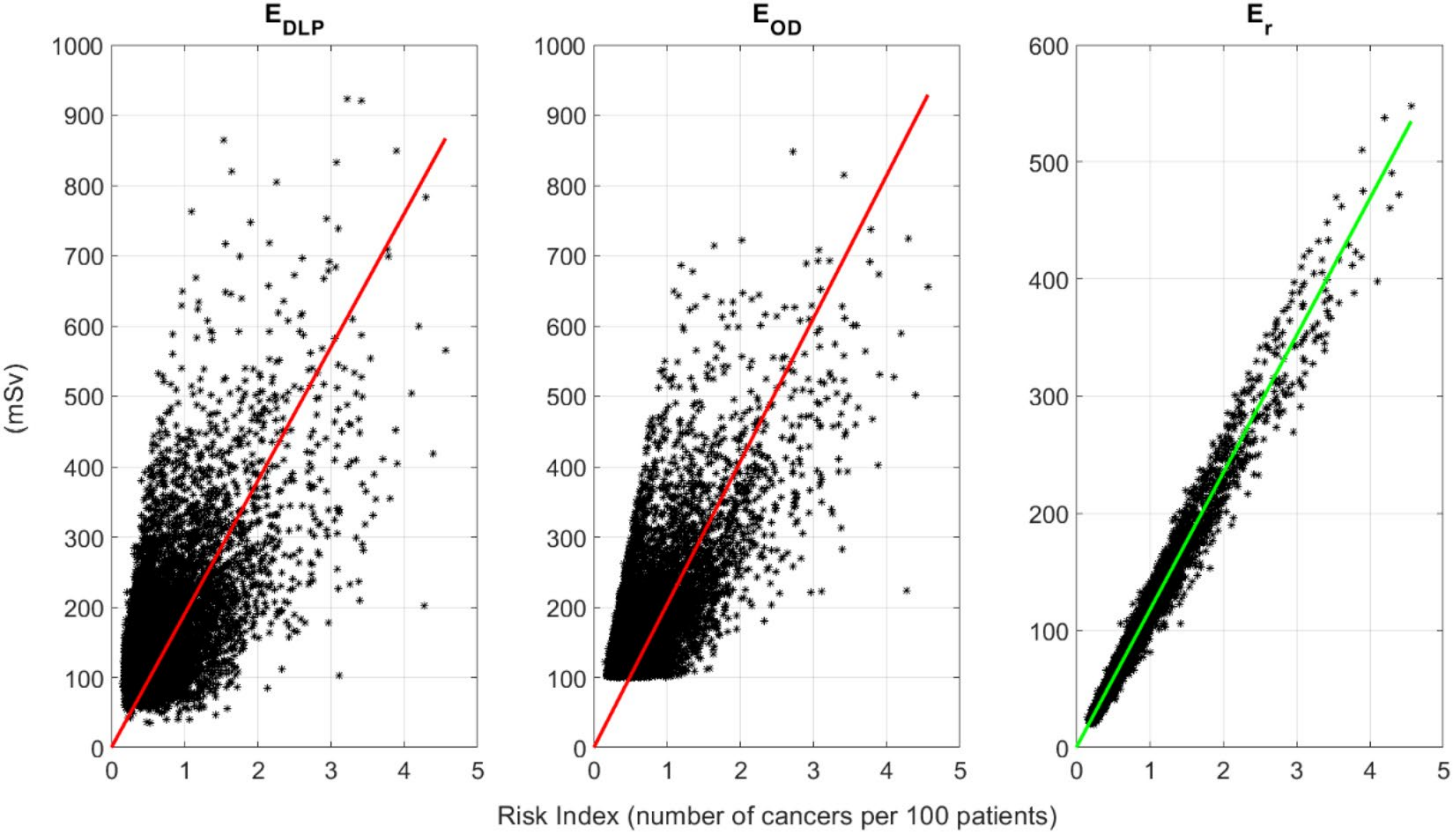
Linear regressions of ${E}_{DLP}$, ${E}_{OD}$, and ${E}_{r}$ with $RI$ assuming null intercept. The relative effective dose curve is green to indicate the best agreement with the risk index.

As reported in Table 4, ${E}_{r}$ showed the best agreement with $RI$ (R^2^ = 0.97) with ${E}_{DLP}$ and ${E}_{OD}$ showing very low R^2^ values. The newly defined relative effective dose, also showed the best risk sensitivity index and risk differentiability index with the DLP-based effective dose showing the poorest RDI and, along with Organ dose-based effective dose, the lowest RSI. The linear regressions are displayed in Fig. 2.

**Table 4:** Fit parameters, risk sensitivity index (RSI), and risk differentiability index (RDI) across the three effective dose computation methods considered in the study.

	E_DLP_	E_OD_	E_r_
Slope (mSv/number of cancers per 100 patients)	189.89	203.50	117.14
R^2^	*0.01*	0.09	**0.97**
Normalized RMSE	*0.55*	0.47	**0.12**
RSI	*0.81*	*0.81*	**0.99**
RDI (number of cancers per 100 patients)	*0.48*	0.41	**0.08**

Bold text indicated the best agreement and italic indicates the poorest.

## Discussion

In this study we applied the calculation of the updated relative effective dose (${E}_{r}$)^10^ to a specific group of about 9000 patients who underwent multiple CT imaging scans cumulating at least 100 mSv of organ dose-based effective dose (${E}_{OD}$) estimated according to the standard ICRP 103 publication definition^6^. We also tested how ${E}_{r}$ depicts risk compared to Risk Index ($RI$), as opposed to depictions provided by ${E}_{OD}$ and ${E}_{DLP}$. The presented results confirmed that ${E}_{r}$ shows better risk characterization performance, both in terms of risk sensitivity (RSI) and differentiability (RDI). This is not surprising as relative effective dose already incorporates age- and sex-specific factors. Moreover, the results show how different effective dose definitions lead to differing radiation risk characterizations and association conclusions, and they do not convey the same insight into the data or a clinical operation.

While the discussion about the use of effective dose for medical exposure remains vibrant, some uses share the consensus of the scientific community^9,14^. First, effective dose, as formally defined, is a risk-relevant quantity drawn from large population, that can be used to compare exposures from different radiological exams, and for education and training of healthcare professionals. Second, it can be used to establish ranges of doses for different diagnostic procedures and implement radiation protection actions. Nonetheless, the effective dose calculation, as defined, relies on tissue detriment effects that are averaged across sexes and ages. Such a fundamental attributes influences the very utility of effective dose for the aforementioned uses. How the appropriateness or safety of a procedure for a patient can be determined if the metric used for that determination is not accurately related to the radiation safety of the exam for that individual? This dilemma is further confused by the modern approaches to patient dose monitoring that calculate organ and tissue absorbed doses using sex- and age-specific anthropomorphic virtual phantoms^15–17^, generating an *effective dose* that neither meets the original definition of the concept nor accounts for sex and age effects^7^.

The proposed ${E}_{r}$ addresses this fundamental challenge. The concept of relative effective dose accommodates the individualization of the dose estimate such that any aforementioned uses can be based on analyses and aggregates of individually-relevant data. It is further in line with the provisions of ICRP, noted earlier^9^. Moreover, the relative effective dose, while more accurately reflective of radiation risk, does not create the “spurious sense of accuracy” that the quantity risk index indicates, as observed by ICRP^9^. ${E}_{r}$, avoiding the use of a unit in terms of incidence or mortality of cancer, sidesteps in projecting a false sense of certainty, sensibly acknowledging the approximate nature of risk estimation.

Comparing different methods of calculating effective dose, this study reconfirmed earlier studies that show how different methods lead to different depictions of population risk. Therefore, any claim about effective dose values and trends, should be always followed by the adopted calculation method description in order to avoid comparisons between distributions coming from different type of calculations. These findings are consistent with a 2018 recommendation from the International Atomic Energy Agency that highlighted how all dose quantities can relate to radiation dose falling into a relevance hierarchy^18^. In particular, ${E}_{DLP}$ relies on CT device output that is converted to a risk surrogate simply by the application of a body region conversion factor. ${E}_{OD}$ combines organ sensitivities, with ${E}_{r}$ finally including also age and sex factors.

The presented results, can also be interpreted from the perspective of radiation protection principles. In particular, radiation metrics based on device output (i.e., CTDI_vol_) is helpful for radiation protection or optimization purposes. However, care should be taken because tube current and kV in some modern machines get modulated, both influenced by patient body habitus, and may cause those metrics to have a non-linear relationship with the radiation exposure to the individual patient. Moreover, the justification of radiological procedures can be better informed by individual relative effective dose (E_r_). For instance, an exam might be justified for and elderly but not a pediatric case though both share the same conventional effective dose. Analogously, the effective dose associated with the same exam for patient of different sex can be similar, whereas E_r_ can better describe the procedure risk also considering the sex of the patient, thus potentially leading to different justification conclusions.

This study was limited only to adult populations; future investigation should try to extend the application of ${E}_{r}$ also to pediatric patients. Moreover, all subjects of the study were from a single institution. Even though the presented results are not affected by differences in clinical practices, the inclusion of different healthcare institutions can be beneficial for a comprehensive evaluation of the proposed new metric. Analogously, the ${E}_{r}$ calculation is based on sex- and age- specific lifetime attributable risk of cancer incidences in the US population reported in BEIR VII report. If risk factors for different populations are available, they can be incorporated in the presented model without changing the general framework. Furthermore, the proposed approach relies on patient-specific organ doses calculated with Monte Carlo methods which are not always available at the point of care. Future extension of the presented methodology can consider its application to large patient populations to extrapolate adjustment or conversion factors also for other metrics, such as the size-specific dose estimate^3,4^, that do not require the implementation of Monte Carlo methods. Finally, our study was based on a series of representative patient models. These models are always advancing. The presented methodology can easily be extended to future representative patient models.

## Conclusion

A person’s age, sex, and size have notable influences on his or her radiation exposure when undergoing a radiological procedure. Current metrics to quantify and to qualify that burden are far short of faithfully representing it. This study showed that a new metric, relative effective dose (${E}_{r}$), offers a radiation risk depiction to an individual consistent with current scientific knowledge, while taking advantage of the established framework and concept of effective dose.

## Data Availability

All data generated and analyzed in the study are available from the corresponding author on reasonable request.
